# Supplementary material for: Evaluation of a New Extracorporeal CO2 Removal Device in an Experimental Setting
Source: Membranes (Basel). 2020 Dec 23;11(1):8. doi: 10.3390/membranes11010008 (PMC7823796; doi:10.3390/membranes11010008)
Supplement: Supplementary file 1 [file membranes-11-00008-s001.pdf]

Supplementary Materials

Evaluation of a New Extracorporeal CO_2 Removal Device in an Experimental Setting

Matteo Di Nardo [1,*], Filippo Annoni [2], Fuhong Su [2], Mirko Belliato [3], Roberto Lorusso [4], Lars Mikael Broman [5,6], Maximilian Malfertheiner [7], Jacques Creteur [2] and Fabio Silvio Taccone [2]

1 Pediatric Intensive Care Unit, Bambino Gesù Children's Hospital, IRCCS, 00165 Rome, Italy
2 Department of Intensive Care, Hôpital Erasme, Université Libre de Bruxelles (ULB), 1050 Brussels, Belgium; filippo.annoni@erasme.ulb.ac.be (F.A.); sufuhong@yahoo.com (F.S.); jacques.creteur@ulb.ac.be (J.C.); fabio.taccone@ulb.ac.be (F.T.)
3 Anestesia e Rianimazione II Cardiopolmonare, Foundation IRCCS, Policlinico San Matteo, 27100 Pavia, Italy; m.belliato@gmail.com
4 Heart & Vascular Centre, Maastricht University Medical Centre, 6229 Maastricht, The Netherlands; roberto.lorussobs@gmail.com
5 ECMO Centre Karolinska, Karolinska University Hospital, 17164 Solna,Stockholm, Sweden; lars.broman@sll.se
6 Department of Physiology and Pharmacology, Karolinska Institutet, 171 77 Stockholm, Sweden
7 Internal Medicine II, University of Regensburg, 93053 Regensburg, Germany; maxmalfertheiner@gmail.com
* Correspondence: matteo.dinardo@opbg.net

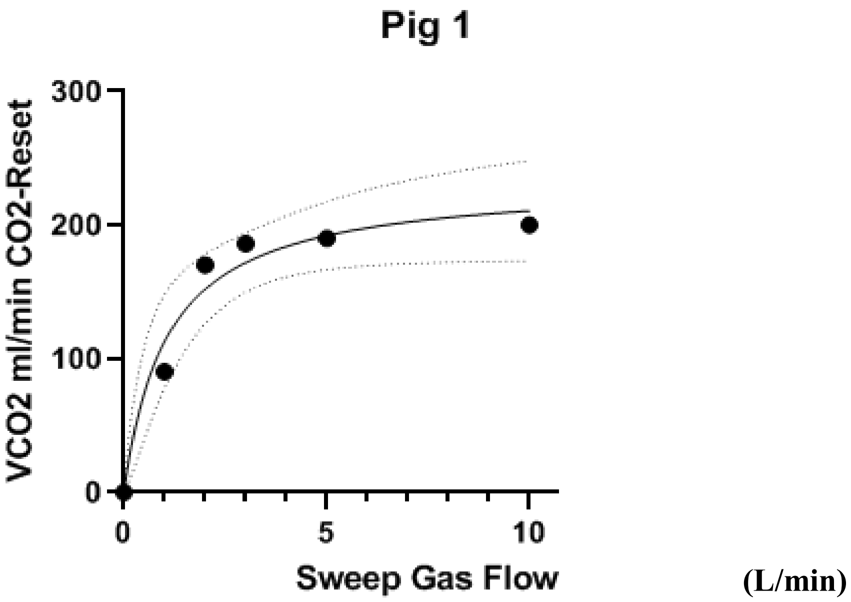

Figure S1. VCO_2 removal at different gas flow (Pig 1).

Table S1. Pig 1: main physiologic variables at baseline (Time 1) and at the beginning of the experiment (Time 2).

	Time 1	Time 2
Respiratory rate (breaths/min)	17	9
Tidal volume-pig (mL)	430	216
Minute ventilation (L/min)	7.31	1.94
Positive end-expiratory pressure (cmH_2O)	5	5
Compliance respiratory system (cmH_2O)	36	30
Respiratory system mechanical power (J/min)	10	1.80
Heart rate (beats/min)	65	70
Central venous pressure (mmHg)	6	6
Mean systemic arterial pressure (mmHg)	88	87
Arterial lactates (mmol/L)	0.90	0.88

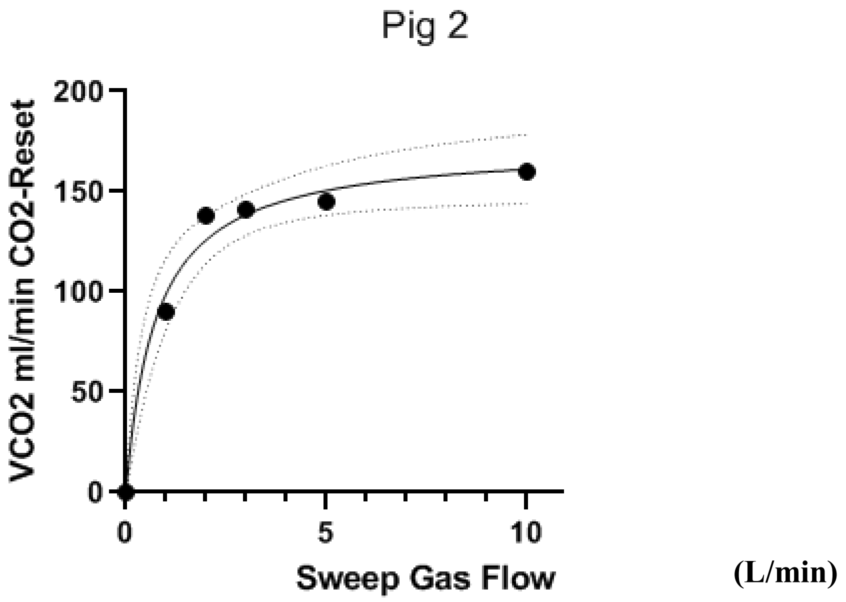

Figure S2. VCO_2 removal at different gas flow (Pig 2).

Table S2. Pig 2: main physiologic variables at baseline (Time 1) and at the beginning of the experiment (Time 2).

	**Time 1**	**Time 2**
Respiratory rate (breaths/min)	20	9
Tidal volume-pig (mL)	400	200
Minute ventilation (L/min)	8	1.8
Positive end-expiratory pressure (cmH_2O)	5	5
Compliance respiratory system (cmH_2O)	28	20
Respiratory system mechanical power (J/min)	13.5	1.92
Heart rate (beats/min)	73	77
Central venous pressure (mmHg)	8	9
Mean systemic arterial pressure (mmHg)	92	93
Arterial lactates (mmol/L)	1.20	1.35

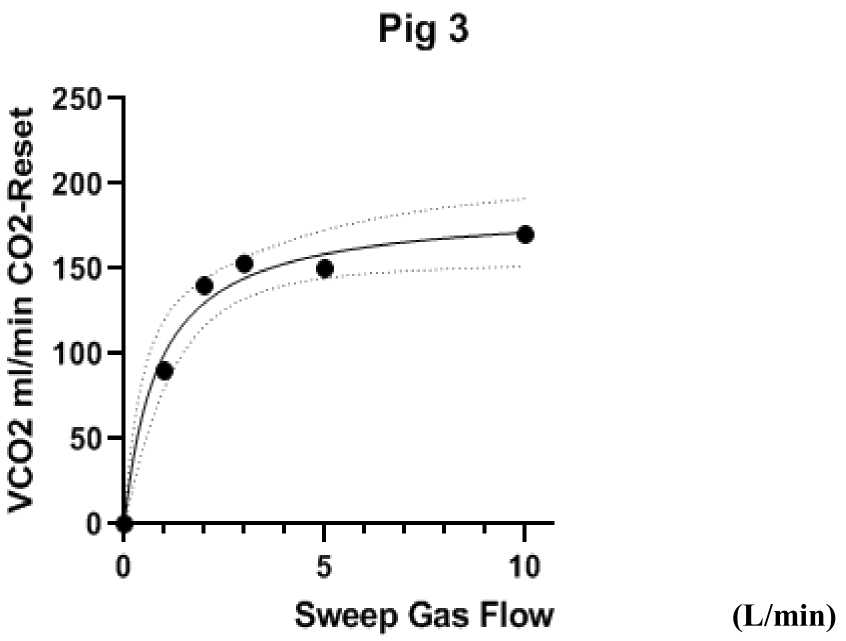

Figure S3. VCO_2 removal at different gas flow (Pig 3).

Table 3. Pig 3: main physiologic variables at baseline (Time 1) and at the beginning of the experiment (Time 2).

	**Time 1**	**Time 2**
Respiratory rate (breaths/min)	18	9
Tidal volume-pig (mL)	450	224
Minute ventilation (L/min)	12.18	2.02
Positive end-expiratory pressure (cmH_2O)	5	5
Compliance respiratory system (cmH_2O)	34	28
Respiratory system mechanical power (J/min)	12.18	1.9
Heart rate (beats/min)	85	81
Central venous pressure (mmHg)	10	10
Mean systemic arterial pressure (mmHg)	100	99
Arterial lactates (mmol/L)	1.60	1.55
